# Ex Vivo Microsuturing of the Human Anterior Lens Capsule Using 10-0 Sutures: A Technical Feasibility Report

**DOI:** 10.7759/cureus.83028

**Published:** 2025-04-26

**Authors:** Joobin Khadamy, Pontus Lithén

**Affiliations:** 1 Ophthalmology, Skellefteå Eye Clinic, Skellefteå, SWE; 2 Ophthalmology, University Hospital of Umeå, Umeå, SWE

**Keywords:** anterior lens capsule, biological graft, in vitro model, lens capsule transplantation, ocular surface reconstruction, ophthalmic suturing, pseudoexfoliation syndrome, regenerative ophthalmology, suture biomechanics, trypan blue staining

## Abstract

The human lens capsule is a robust, transparent basement membrane commonly discarded during cataract surgery. Although its use as a biological graft has gained interest in ocular surgery, practical methods for securing the capsule, such as suturing, have not been evaluated. This technical report presents the first in vitro assessment of anterior lens capsule sutureability using fine 10-0 sutures.

Anterior capsule specimens were obtained from two patients with pseudoexfoliation syndrome undergoing routine cataract surgery. After being stored for four hours in balanced salt solution (BSS), the specimens, one stained with trypan blue and the other unstained, were spread on a plastic surface (a cellophane sheet: as a non-biologic backing substrate) and sutured using 10-0 nylon (Ethilon™, Ethicon, Raritan, NJ, USA) and 10-0 polyglactin 910 (Vicryl™, Ethicon, Raritan, NJ, USA) under an operating microscope. Both suture types could be successfully passed through the capsule without immediate tearing. The stained capsule enhanced visibility but appeared slightly more fragile, while the unstained capsule demonstrated greater elasticity. Attempts to reapproximate full-thickness capsule tears using sutures were unsuccessful.

Viscoelastic agents, including MiniVisc® Plus (Bausch + Lomb, Vaughan, Canada) and DisCoVisc® (Alcon Laboratories, Fort Worth, TX, USA), did not aid in capsule stabilization and reduced visibility during manipulation. No specialized instruments beyond standard ophthalmic tools were used.

These findings demonstrate that human anterior lens capsules can tolerate fine suturing in vitro and suggest their potential as suturable biological grafts in applications such as corneal patching, pterygium surgery, or ocular surface reconstruction. However, limitations in tear repair and in vivo behavior require further investigation to establish clinical utility.

## Introduction

The anterior lens capsule is the thickest basement membrane in the human body, composed primarily of type IV collagen, laminin, and other extracellular matrix proteins [1,2]. It forms a transparent yet durable envelope around the crystalline lens and plays a critical role in maintaining the immune privilege of the intraocular environment. Despite its biological resilience, the anterior capsule is routinely removed and discarded during cataract surgery. In recent years, however, it has attracted growing interest as a biocompatible graft or scaffold in ocular surgery and regenerative medicine [3].

A recent review has summarized multiple experimental and clinical applications of the lens capsule [3], including its use as an autologous patch in refractory macular holes and optic disc pit maculopathy, a substitute for Bowman's layer in corneal ulcer repair, and a scaffold for corneal endothelial and limbal stem cell cultivation [3]. Additionally, anterior capsule grafts placed under the scleral flap during trabeculectomy have shown promise as biologically derived adjuncts to modulate postoperative healing and improve bleb function [4,5]. Moreover, it has a potential role as a carrier for drug delivery or stem cell transplantation in regenerative surgery [3]. These diverse applications underscore the capsule's potential as a transparent, immune-privileged, and resilient biomaterial.

Most clinical uses of the lens capsule have avoided direct suturing [3]. Instead, fixation has typically relied on tamponade agents such as silicone oil or air in macular surgery [6], overlay with bandage contact lenses in corneal surface reconstruction [7], or mechanical compression beneath a scleral flap in glaucoma filtration surgery [4,5]. In some instances, stabilization has been achieved using tissue adhesives like cyanoacrylate or fibrin glue or through the use of autologous blood coagulum [7-9]. While these techniques can be effective in specific scenarios, they offer limited control over graft positioning, especially in mobile or irregular surgical fields.

Although remnants of the lens capsular bag have been sutured in anchoring intraocular lenses (IOLs) during scleral fixation, the feasibility of placing microsurgical sutures directly through capsule tissue for graft fixation has not been rigorously evaluated [3]. Demonstrating that the capsule can accommodate fine sutures would open new possibilities for precisely securing grafts in anterior segment surgeries. Conversely, if suturing causes capsule fragmentation or failure, alternative fixation strategies would be required for reliable use.

In this context, we performed a bench-top experiment to evaluate the sutureability of human anterior lens capsules using 10-0 ophthalmic sutures. We assessed the capsule's resistance to tearing, suture passage behavior, and handling characteristics under different staining and hydration conditions. To our knowledge, this is the first technical report to systematically evaluate the mechanical tolerance of the human lens capsule tissue to suturing, providing early insights into its future use as a suturable graft in ophthalmic surgery.

## Technical report

Specimen collection

Two anterior lens capsule specimens were obtained from separate patients with pseudoexfoliation syndrome (PEX) during routine phacoemulsification cataract surgery. In both cases, a continuous curvilinear capsulorhexis (approximately 5 mm in diameter) was performed using standard capsulorhexis forceps, yielding a circular segment of the anterior capsule. Each excised specimen exhibited a curvilinear tear extending from the central capsulorhexis punch to the periphery, likely due to intraoperative manipulation.

This study was reviewed by the local ethics committee of Västerbotten Eye Clinic, Skellefteå, Sweden. As the project consisted of a technical case report involving only discarded, anonymized anterior lens capsule specimens from routine cataract surgeries and did not involve live tissue, human subjects, or identifiable patient information, a formal ethics approval number was not required according to institutional and regional guidelines. Both patients had provided informed consent for the use of their discarded surgical tissue in laboratory testing and for the publication of anonymized data, including images and video. All procedures complied with the Declaration of Helsinki and the European Union General Data Protection Regulation (EU GDPR 2016/679).

Immediately after extraction, the capsule specimens were placed in a sterile balanced salt solution (BSS) and stored at room temperature for up to four hours before testing. One capsule had been stained intraoperatively with trypan blue for routine visualization; the other remained unstained. No decellularization, enzymatic treatment, or additional manipulation was performed beyond routine surgical care. Fibrillar pseudoexfoliative deposits were noted on the capsule surfaces but did not interfere with specimen handling. Residual lens epithelial cells and surface material were not intentionally removed before testing.

All specimen handling and storage adhered to standard operating room procedures. After testing, the specimens were discarded per institutional biowaste protocols. No deviation from clinical care occurred, and no patient-identifiable data were retained.

Suturing setup and materials

In vitro suturing was performed on a clean, flat plastic surface under high magnification using the ZEISS OPMI LUMERA 700 surgical microscope (Carl Zeiss Meditec AG, Jena, Germany), simulating the scale and conditions of intraocular surgery. A sheet of transparent cellophane packaging material served as the backing surface, providing a smooth and slightly adherent platform ideal for stabilizing the delicate anterior lens capsule. Each capsule was gently unrolled and flattened onto this surface.

Standard microsurgical instruments routinely employed in cataract surgery were used throughout the procedure. These included fine capsulorhexis or tying forceps for handling both the capsule and the sutures, a microsurgical needle holder for precise manipulation, Westcott scissors as needed, and sterile gauze for fluid blotting.

**Figure 1:**
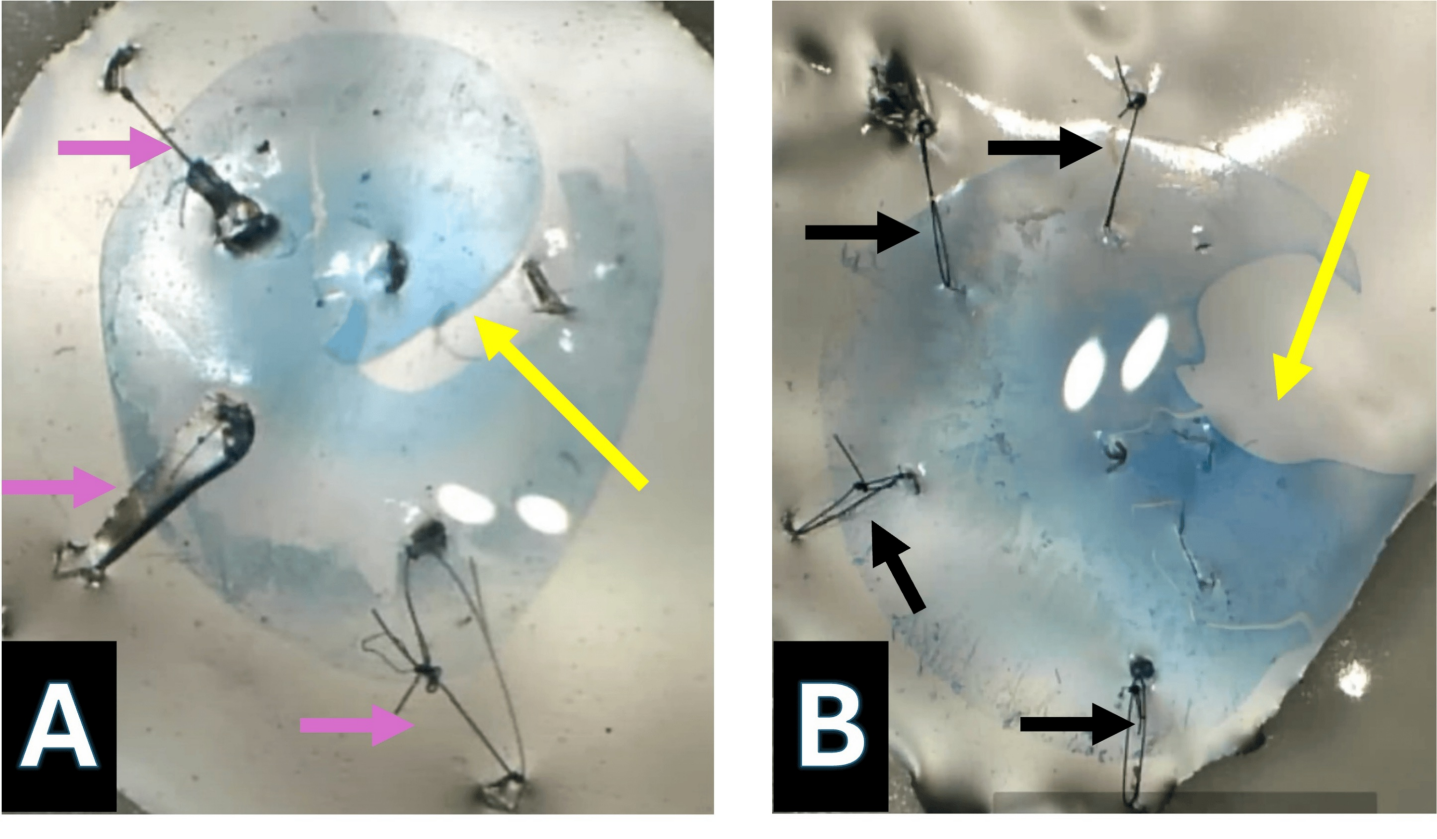
In vitro suturing of anterior lens capsules using 10-0 Vicryl and nylon sutures. (A) Capsule A with three 10-0 polyglactin 910 (Vicryl™, Ethicon, Raritan, NJ, USA) sutures (purple arrows) placed at the capsule margins. The capsule was stained intraoperatively with 0.06% trypan blue to enhance visualization. The yellow arrow indicates a tear created during routine capsulorhexis using capsulorhexis forceps. (B) Capsule B with four 10-0 polyamide (Ethilon™, Ethicon, Raritan, NJ, USA) sutures (black arrows) placed peripherally. The capsule was initially unstained but later stained with trypan blue due to difficulty in visualization. The yellow arrow highlights a capsular tear incurred during intraoperative manipulation with capsulorhexis forceps. Both capsules were stabilized on a transparent cellophane sheet and sutured under high magnification using the ZEISS OPMI LUMERA 700 microscope (Carl Zeiss Meditec AG, Jena, Germany).

Two suture types were evaluated. The first was 10-0 polyglactin 910 (Vicryl™, Ethicon, Raritan, NJ, USA), a braided absorbable suture mounted on a spatulated micropoint needle. The second was 10-0 polyamide/nylon (Ethilon™, Ethicon, Raritan, NJ, USA), a monofilament non-absorbable suture on a similarly small needle. Both suture types are commonly used in delicate ophthalmic procedures such as conjunctival and corneal wound closure. Capsule A was sutured using Vicryl and Capsule B with nylon (Figure 1). A direct comparison was not intended; only one type of suture was placed in each capsule, limiting the ability to control for potential variability in tissue characteristics. Ideally, at least one suture of each type should have been placed in both capsules to mitigate the effect of inter-sample differences on subjective handling experience.

Trypan blue staining, routinely used in cataract surgery to assist capsulorhexis, was anticipated to similarly aid visualization during suturing without significantly altering the biomechanical properties of the tissue. The dye used was VisionBlue® (Dutch Ophthalmic Research Center (DORC), Zuidland, Netherlands). Capsule A was stained intraoperatively with 0.06% trypan blue ophthalmic dye for 30 seconds and then rinsed in BSS, in accordance with routine clinical visualization needs. This imparted a light blue tint to the capsule, enhancing contrast against the background. Capsule B was initially left unstained, as no dye had been used during the original cataract procedure. It served as a control to assess tissue handling and visibility without contrast enhancement. However, due to significant difficulty in visualizing the unstained membrane during manipulation, Capsule B was subsequently stained with trypan blue to complete suturing under improved visualization.

The capsule adhered gently to the plastic sheet after blotting, allowing stable positioning during suturing. A detailed explanation of this adhesion behavior is discussed later. The application of ophthalmic viscoelastic agents, including MiniVisc® Plus (Bausch + Lomb, Vaughan, Canada) and DisCoVisc® (Alcon Laboratories, Fort Worth, TX, USA), did not facilitate capsule stabilization or spreading. On the contrary, these agents impaired visualization and hindered the effective manipulation of the tissue on the plastic surface.

To maintain hydration while preventing flotation, the capsule was kept moist with a minimal amount of BSS. The most stable configuration for suturing was achieved by gently blotting away excess fluid with surgical gauze or a sponge, allowing the capsule to remain slightly damp and softly adherent to the surface. This approach provided optimal conditions for stable and precise microsurgical suturing.

Suture passage and handling

Both stained (Capsule A) and unstained (Capsule B) anterior lens capsules exhibited notable resilience during suture passage. All sutures were placed in visually intact areas, well away from pre-existing peripheral tears, which did not affect handling or knot formation. The 10-0 needles penetrated the capsule without visible resistance or tissue fragmentation. Although biomechanical testing was not performed, the capsule appeared to behave like a thin, elastic film, dimpling slightly on needle entry and returning to shape upon exit, without crumbling or tear propagation. Puncture sites remained localized, and even when needle re-passes were performed at or near previous entries, no tearing or structural compromise was observed under microscopic visualization.

Elastic deformation under traction allowed multiple sutures to be placed in each capsule sample without catastrophic tearing. Knot tying had to be performed delicately; no cheese-wiring of the capsule was observed during suture tightening. However, the primary risk of tearing arose from the sharp, spatulated needle tip, particularly with Vicryl, which could cause entrance-edge tearing if not carefully aligned or if excessive force was used on insertion.

Sutures could be passed in either direction; entering or exiting from the capsule side was technically feasible. However, when exiting on the capsule side, counter-pressure using tying forceps was required to prevent the capsule from unfolding or rolling. This maneuver ensured stable knot placement and minimized the risk of introducing folds or tractional tears during tying.

Both Vicryl and nylon sutures held well, forming secure knots. Sutures could be gently rotated and buried from the capsule surface without inducing damage, further supporting the suture compatibility of the intact anterior capsule tissue under controlled microsurgical conditions.

Capsule integrity and tear resistance

The intact part of the capsules tolerated multiple sutures with minimal deformation and no spontaneous tearing. However, pre-existing full-thickness tears could not be repaired with sutures, and attempts to reapproximate edges failed as sutures pulled through the thin torn margins. Multiple interrupted suturing was found feasible. The capsule tolerated some degree of traction without tearing. 

These findings are demonstrated in Video 1, which captures key aspects of capsule elasticity, suture behavior, hydration effects, and manipulation technique.

**Video 1:** In vitro suturing of the human anterior lens capsule using 10-0 nylon and Vicryl sutures. This video demonstrates the key steps and observations from the ex vivo suturing of human anterior lens capsule specimens. The unstained and trypan blue-stained capsules are unfolded and stabilized on a flat plastic surface using minimal fluid. Interrupted 10-0 polyglactin (Vicryl™, Ethicon, Raritan, NJ, USA) and polyamide (Ethilon™, Ethicon, Raritan, NJ, USA) sutures are placed under high magnification. The intact capsule margin tolerates multiple suture passes without folding, tearing, or structural compromise. Attempts to approximate a full-thickness tear fail, illustrating the capsule's inability to support suturing at ruptured edges. Staining with trypan blue significantly enhances the visualization of the capsule during manipulation. Needle entries from both sides are shown, and successful knot placement requires delicate handling and occasional counter-pressure. Final scenes confirm suture integrity and tissue resilience under mild traction.

Summary of observations and handling characteristics

Our ex vivo evaluation showed that human anterior lens capsule tissue is amenable to microsurgical suturing using 10-0 Vicryl and nylon sutures when handled with care. The capsule's biomechanical response, hydration sensitivity, visualization strategies, and needle interaction were systematically assessed and are summarized in Table 1. Sutures could be successfully placed and secured without cheese-wiring, and the tissue tolerated gentle traction. Trypan blue staining was particularly beneficial in improving visibility without compromising the capsule's integrity.

**Table 1:** Summary of key observations in lens capsule suturing.

Parameter	Observation	Implication
Suture compatibility	10-0 Vicryl and nylon both penetrated and held in intact capsule with secure knots	Microsuturing is feasible on intact anterior lens capsule
Capsule behavior	Dimpled on needle entry, elastic return post-exit; did not tear or propagate holes	Capsule behaves like a resilient elastic membrane
Suture direction	Sutures could enter or exit from capsule side; exiting required counter-hold during tying	Both directions feasible; counter-pressure needed to prevent folding
Knot tying	No cheese-wiring observed; knots tolerated if tension was controlled	Secure throws achievable with gentle handling
Buried/in-rotated sutures	Sutures could be buried or rotated from the capsule side	Provides a low-profile closure option
Needle shape	Smaller wide-spatula needles preferred; sharp spatula tips (esp. Vicryl) occasionally caused entrance tears	Needle geometry influences capsule safety
Suture traction	Once placed, sutures withstood modest traction without tearing	Capsule shows good tensile strength under low tension
Staining with trypan blue	Greatly enhanced visualization without altering mechanical properties	Strongly recommended during capsule manipulation
Hydration (optimal)	Moist, non-submerged state allowed adherence and flexibility	Essential for tissue stability
Hydration (overdry)	Led to brittleness and crumbling of capsule edge	Requires prompt rehydration to restore pliability
Hydration (overwet)	Impaired visualization; capsule became difficult to localize under fluid	Thin fluid film preferred; avoid full immersion
Viscoelastic use	MiniVisc® Plus (Bausch + Lomb, Vaughan, Canada) and DisCoVisc® (Alcon Laboratories, Fort Worth, TX, USA) impaired visibility and handling	Not recommended as a stabilizing agent
Torn capsule behavior	Sutures pulled through thin edges and could not close full-thickness tears	Torn capsules cannot be reliably reapproximated
Capsule size	Each 5 mm disc accommodated 3-5 sutures	Even small capsulorhexis specimens can be sutured effectively

## Discussion

This technical report explores the microsurgical behavior of the human anterior lens capsule in an ex vivo setting, demonstrating its feasibility for future use as a suturable biological graft. The capsule showed elastic deformation during suture passage, did not propagate tears from puncture sites, and tolerated knot tying when handled delicately. These findings offer a practical foundation for expanding the clinical use of lens capsule grafts in ophthalmology, particularly in settings where mechanical fixation is preferred or suture-based control of graft position could offer advantages over other fixation methods.

What distinguishes this report is its direct evaluation of capsule sutureability, a previously untested parameter critical for advancing the capsule from a passive to an actively positionable biological graft. While numerous applications of lens capsule transplantation have been explored in macular holes, optic disc pits, corneal ulcers, and glaucoma bleb reinforcement, virtually all have relied on tamponade, contact lens overlays, tissue adhesives, or compression techniques rather than sutures [3].

One speculative yet clinically plausible direction is the use of sutured anterior capsule grafts in ocular surface reconstruction, such as in pterygium surgery as a substitute for amniotic membrane or conjunctival autograft. A suturable, transparent, basement membrane-like scaffold could offer improved durability and long-term stability, particularly when combined with antifibrotic therapies. Moreover, other vital dyes such as indocyanine green (ICG) may be evaluated in the future for staining [2,10], although trypan blue remains the most established choice.

From a tissue banking and preservation perspective, the use of capsule tissue from patients with PEX underscores the importance of careful donor screening and classification [3]. Although the capsule retained its mechanical integrity after four hours in a BSS medium, it remains unknown whether capsules from eyes with more advanced PEX, fibrotic remodeling, or systemic connective tissue disorders would demonstrate similar suture tolerance or elasticity. Future investigations should assess how disease state, age, or prior ocular surgery affect capsule biomechanics. Inflammatory alterations to the lens capsule, such as those reported during immune cell invasion in uveitic eyes [11], further highlight the importance of donor screening to ensure graft quality and tissue compatibility.

Future studies should also investigate a broader range of microsurgical parameters to better define the capsule's mechanical tolerance and clinical versatility. For example, different needle geometries and materials, such as 10-0 polypropylene or finer nylon gauges, should be assessed for suture behavior and entrance-edge safety. The performance of sutures with varying lengths, tip designs, or curvature radii may also impact outcomes. Additionally, hydrophilic viscoelastic agents like hydroxypropyl methylcellulose (HPMC) should be evaluated for their potential to facilitate intraoperative capsule spreading and stabilization, particularly in the absence of staining. Beyond the anterior capsule, the biomechanical behavior and suture compatibility of the posterior capsule should also be studied, as it may offer different structural responses and clinical applications.

The tractional force tolerance of sutured capsules is another critical parameter for future evaluation, potentially even more relevant than donor age or pediatric tissue origin. While fibrillar composition, defined by collagen IV and extracellular matrix, may vary with systemic or ocular disease, future work should also establish which age groups provide capsules with the most favorable elasticity and tensile integrity. Screening protocols in lens capsule banking may benefit from advanced non-invasive tools such as the Brillouin Optical Scanner System (BOSS) [12], which could quantify viscoelasticity prior to graft use and improve donor selection. Additionally, future studies should evaluate the capsule's potential for biointegration and controlled biodegradability when used as an in vivo graft material, particularly its interaction with host tissue and immune response.

The capsule's spontaneous adhesion to the plastic initially aided by capillary forces in the thin BSS film persisted even after partial desiccation, suggesting that electrostatic or triboelectric attraction may also contribute to stabilization [13]. This behavior may assist intraoperative handling, though it may differ on biologic surfaces such as the conjunctiva or corneal stroma, warranting further testing in more clinically analogous environments.

Limitations of the current work include the small sample size, the lack of quantitative biomechanical testing (e.g., suture pull-out strength or elasticity and traction tolerance), and the absence of in vivo correlation. Suturing was limited to intact capsule regions; attempts to repair large tears failed, underscoring the capsule's inability to support tension at ruptured edges. Furthermore, while both nylon and Vicryl sutures performed well, needle geometry and insertion angle significantly influenced entrance-edge safety, with wider spatulated needles posing more risk of tearing.

Additional unaddressed factors include the potential impact of elapsed time from capsule extraction to suturing, as prolonged intervals might affect capsule fragility. The influence of intraoperative capsule manipulation duration, with extended handling potentially diminishing capsule integrity, was not evaluated. Furthermore, differences between using a cystotome and capsulorhexis forceps for capsule removal could affect the consistency and predictability of the capsule margin. The distribution and density of PEX material on capsules were not systematically assessed, raising questions about whether sutures placed randomly or selectively in PEX-free areas differ in outcomes.

Future studies should assess the in vivo healing response to sutured capsule grafts, including fibrosis, biodegradation, and tissue integration. Comparative studies versus amniotic membrane and other ocular surface materials would help define the capsule's specific role in ocular surface and anterior segment reconstruction. Standardizing donor preparation, sterilization, and potential banking protocols will also be critical for broader adoption [3].

The present report adds a practical step toward clinical translation by confirming that suturing is technically feasible and does not cause immediate capsule distortion. Beyond suturing, alternative fixation strategies such as cyanoacrylate glue, tissue adhesives, or laser bonding should be explored [14].

## Conclusions

The human anterior lens capsule is a mechanically resilient tissue that can be sutured ex vivo with fine microsurgical sutures under ophthalmic surgical conditions. This capability expands its potential as a graft in anterior segment reconstruction and other applications where secure fixation is needed, particularly given that all procedures in this study were completed using only standard microsurgical instruments available in routine cataract surgery. While further validation is required for in vivo use, these findings establish a foundational step toward repositioning the lens capsule from the discarded tissue to a suturable, biologically compatible surgical material. This work provides a foundation for incorporating the lens capsule into future suture-based ocular surface repair strategies.
